# Gait characterization in golden retriever muscular dystrophy dogs using linear discriminant analysis

**DOI:** 10.1186/s12891-017-1494-4

**Published:** 2017-04-12

**Authors:** Bodvaël Fraysse, Inès Barthélémy, El Mostafa Qannari, Karl Rouger, Chantal Thorin, Stéphane Blot, Caroline Le Guiner, Yan Chérel, Jean-Yves Hogrel

**Affiliations:** 1 Atlantic Gene Therapies, INSERM UMR 1089, Nantes, France; 2 INSERM U955-E10 Biology of the NeuroMuscular System, 94000 Créteil, France; 3 Université Paris-Est, École Nationale Vétérinaire d’Alfort, 94700 Maisons-Alfort, France; 4 Faculté de Médecine, 94000 Créteil, France; 5 LUNAM University, ONIRIS, National College of Veterinary Medicine, Food Science, and Engineering, USC “Sensometrics and Chemometrics Laboratory”, Nantes, France; 6 Atlantic Gene Therapies, INRA UMR 703, ONIRIS, Nantes, France; 7 Nutrition and Endocrinology Unit, ONIRIS, National College of Veterinary Medicine, Food Science, and Engineering, Nantes, France; 8 Neuromuscular Physiology and Evaluation Lab, Institute of Myology, Paris, France

**Keywords:** Muscular dystrophy, GRMD, Treatment evaluation, Gait assessment, Discriminant analysis, Animal model, Accelerometry

## Abstract

**Background:**

Accelerometric analysis of gait abnormalities in golden retriever muscular dystrophy (GRMD) dogs is of limited sensitivity, and produces highly complex data. The use of discriminant analysis may enable simpler and more sensitive evaluation of treatment benefits in this important preclinical model.

**Methods:**

Accelerometry was performed twice monthly between the ages of 2 and 12 months on 8 healthy and 20 GRMD dogs. Seven accelerometric parameters were analysed using linear discriminant analysis (LDA). Manipulation of the dependent and independent variables produced three distinct models. The ability of each model to detect gait alterations and their pattern change with age was tested using a leave-one-out cross-validation approach.

**Results:**

Selecting genotype (healthy or GRMD) as the dependent variable resulted in a model (Model 1) allowing a good discrimination between the gait phenotype of GRMD and healthy dogs. However, this model was not sufficiently representative of the disease progression. In Model 2, age in months was added as a supplementary dependent variable (GRMD_2 to GRMD_12 and Healthy_2 to Healthy_9.5), resulting in a high overall misclassification rate (83.2%). To improve accuracy, a third model (Model 3) was created in which age was also included as an explanatory variable. This resulted in an overall misclassification rate lower than 12%. Model 3 was evaluated using blinded data pertaining to 81 healthy and GRMD dogs. In all but one case, the model correctly matched gait phenotype to the actual genotype. Finally, we used Model 3 to reanalyse data from a previous study regarding the effects of immunosuppressive treatments on muscular dystrophy in GRMD dogs. Our model identified significant effect of immunosuppressive treatments on gait quality, corroborating the original findings, with the added advantages of direct statistical analysis with greater sensitivity and more comprehensible data representation.

**Conclusions:**

Gait analysis using LDA allows for improved analysis of accelerometry data by applying a decision-making analysis approach to the evaluation of preclinical treatment benefits in GRMD dogs.

## Background

Duchenne muscular dystrophy (DMD) is an X-linked disorder caused by various mutations in the gene encoding for dystrophin, resulting in the absence of the functional protein in muscle fibres [1]. DMD patients display progressive muscle weakness leading to the loss of independent mobility in young adolescents and respiratory and heart failure in young adults. While gene, cell, and pharmacological therapies have all been investigated [2, 3], there is currently no curative therapy for DMD. However, several issues such as efficacy of studied drugs, locoregional or systemic medication pathways or dosage, remain to be further explored. The preclinical DMD model of choice is the dystrophin-deficient golden retriever muscular dystrophy (GRMD) dog, which closely mimics many aspects of the human disease [4, 5]. Scale is a key factor that influences the translation of data from animal models to humans. Thus, when studying mechanical impacts, molecular diffusion and/or cell migration data acquired in GRMD dogs is much more relevant to human DMD than that obtained in smaller animal models such as the mdx mutant mouse [6]. The downside of the GRMD model is that it is more expensive to purchase and house, and the use of "man's best friend" for research purposes entails additional political and ethical considerations. Accordingly, limited numbers of GRMD dogs are generally used in studies. At first glance, this appears to be a major impediment to the preclinical evaluation of therapies, particularly given that GRMD dogs exhibit considerable inter-individual phenotypic variability [6–8]. However, there are numerous similarities between canine and human diseases, and considerable inter-individual variability is also observed among human patients [9]. Moreover, given the rarity of muscular dystrophies, and for obvious ethical reasons, clinical studies in man generally involve limited numbers of patients and pose similar challenges to those performed in GRMD dogs. Tools that allow the establishment of better readouts of disease progression and treatment response are essential to overcome limitations imposed by small sample sizes and wide inter-individual variability in studies using the GRMD model, and to better predict the pathogenesis of muscular dystrophy and treatment efficacy in humans.

Studies by several groups have conducted gait analysis in GRMD dogs [10–14]. Using accelerometry analysis in these animals we have demonstrated less regular and less powerful acceleration, decreased stride length and frequency, and a redistribution of power from the cranio-caudal to the medio-lateral axis [11]. Using the main gait variables, we developed a global gait index that consistently detected early changes in gait patterns in GRMD, as well as the progressive deterioration of gait quality. This index was based on the use of principal component analysis (PCA) and the computation of Euclidean distances at multiple time points with respect to an age-matched control group [10]. Given the inherent complexity of the methodology, we believe that this approach is not best suited to the problem at hand, and in fact may hinder the evaluation of therapies in preclinical studies in GRMD dogs. To increase sensitivity and aid interpretation of the outcomes, we sought to design a simpler and more appropriate analytical method using linear discriminant analysis (LDA). Like PCA, LDA is an orthogonal transformation and data reduction technique, but unlike PCA, LDA seeks to minimize intra-group variance and maximize inter-group variance. Moreover, LDA yields a predictive model based on control group data. This is achieved by computation of group membership based on experimental data and assignment rules, which allow the prediction of group membership for future observations. Here, we describe a new method of 3D accelerometric gait analysis using LDA. We discuss the choice of dependent and independent variables and describe how to represent the results in a manner that can be understood by a broad range of users, including those unfamiliar with LDA. Finally, we demonstrate the validity of this method by reanalysing data from a previous study regarding immunosuppressive treatment in GRMD dogs.

## Methods

### Subjects

All procedures were carried out in accordance with Guidelines for the Care and Use of Laboratory Animals, and approved by the common Ethical Committee of the National Veterinary School of Alfort, ANSES, and UPEC. Eight healthy golden retrievers and 20 GRMD dogs (all males) were included in the study. All animals came from the French GRMD colony. The healthy dogs were littermates of some of the GRMD dogs used. Six of the healthy dogs and 12 of the GRMD dogs had participated in a previous study [10]. All dogs were housed in the same facilities, and were genotyped as previously described [15]. Only the GRMD dogs that were still ambulatory after 9 months of age were included in this study.

### Evaluation of gait quality using 3D accelerometry

As previously described [11], the 3-dimensional accelerometer recorder used was a Locometrix® gait analysis system, composed of three orthogonally positioned accelerometers, which can record accelerations along the dorso-ventral, cranio-caudal, and medio-lateral axes.

### Immunosuppressed dogs

To assess the efficiency of our method, we analysed gait data acquired for four immunosuppressed dogs that had been published in a previous paper [12]. These dogs had been treated with high doses of oral prednisolone (2 mg/kg/d) and cyclosporine A (initial dose of 20 mg/kg/d) between 2 and 9 months of age.

### Gait testing

Dogs were carried from the kennel to a 45-meter-long testing corridor located close to the laboratory facilities, as previously described [10]. The belt to which the accelerometric device was attached was fastened around the thorax of the dog, near the centre of gravity at rest. Each animal was tested twice per month from 2 months of age (when motor clinical signs appear in animals that survive the neonatal period) to 9.5 months of age, thus covering the period of growth and disease progression [4]. To enhance the discriminatory power of the method, values obtained for GRMD dogs that survived to 12 months were also included in the analysis. Young puppies were familiarized with the corridor and the belt before the test. The height at withers (HW) was measured at the end of each test. All tests were performed by the same experimenter (IB). In each test, the dog was encouraged to walk or run at its preferred gait, and its speed calculated over a distance of five meters, as previously described [11].

### Data analysis

For quadrupedal gait analysis, acceleration curves were analysed using the software provided by the manufacturer of the recording device (Equimetrix®, Centaure Metrix, Evry, France). A 10-second sequence of steady-state locomotion, which was easily identifiable in the dorso-ventral acceleration curves, was analysed. The following variables, which have been previously described in detail [10, 11], were computed: stride frequency (SF, /s), stride regularity (Reg, dimensionless), total power of accelerations (TP, W/kg), relative components of the total power along the three axes (%) (calculated by dividing cranio-caudal (CCP), dorso-ventral (DVP), or medio-lateral power (MLP) by total power (TP)), and stride length (calculated by dividing the speed by SF), which was normalized to height at withers (SL/HW) in order to circumvent the effect of limb length on this variable. For the sake of consistency, only observations with a Reg value >70 were considered for analysis. Gait testing consisted of two consecutive round trips in the corridor. However, in contrast to healthy dogs, it was very difficult, if not impossible, for some GRMD dogs to complete the second round trip.

### Discriminant analysis

The main objective of the present study was to assess the capacity of discriminant analysis (DA) to evaluate gait, detect functional alterations, and evaluate treatment benefits during the growth period in GRMD dogs. We used gait data obtained from healthy and GRMD dogs and performed DA using XLSTAT software (Addinsoft™). This Excel add-in extends the analytical functions of Excel and covers the key requirements for data analysis and statistics. DA is a commonly used multivariate data analysis method. The aim of this supervised method is to predict group memberships of a set of individuals based on multivariate data. In this scenario, the groups of individuals are assumed to be known a priori. DA reduces the dimensionality of the data at hand by computing synthetic variables, often called canonical variables or factors, the aim of which is to maximize inter-group variance while minimizing intra-group variance. DA yields new variables, which are linear combinations of the original variables. The maximum number of such variables is equal to the number of groups minus one and are usually noted F1 to F(n-1) where n is the number of groups. However, in practice, only the first few canonical variables are used for the purpose of discrimination, since the remaining canonical variables may be predominantly associated with noise present in the data. The graphical displays generated using the retained canonical variables are useful to depict the separation of groups. DA methods include linear DA (LDA) and quadratic DA (QDA) [16]. LDA is a parametric method that assumes Gaussian distributions with the same variance-covariance matrix within the various groups. In practice however, this is often not the case. Nonetheless, even in the case of a slight deviation from this requirement, LDA performs reasonably well [17], and was thus the method selected for the present study.

## Results

### Discriminant analysis

**Fig. 1:**
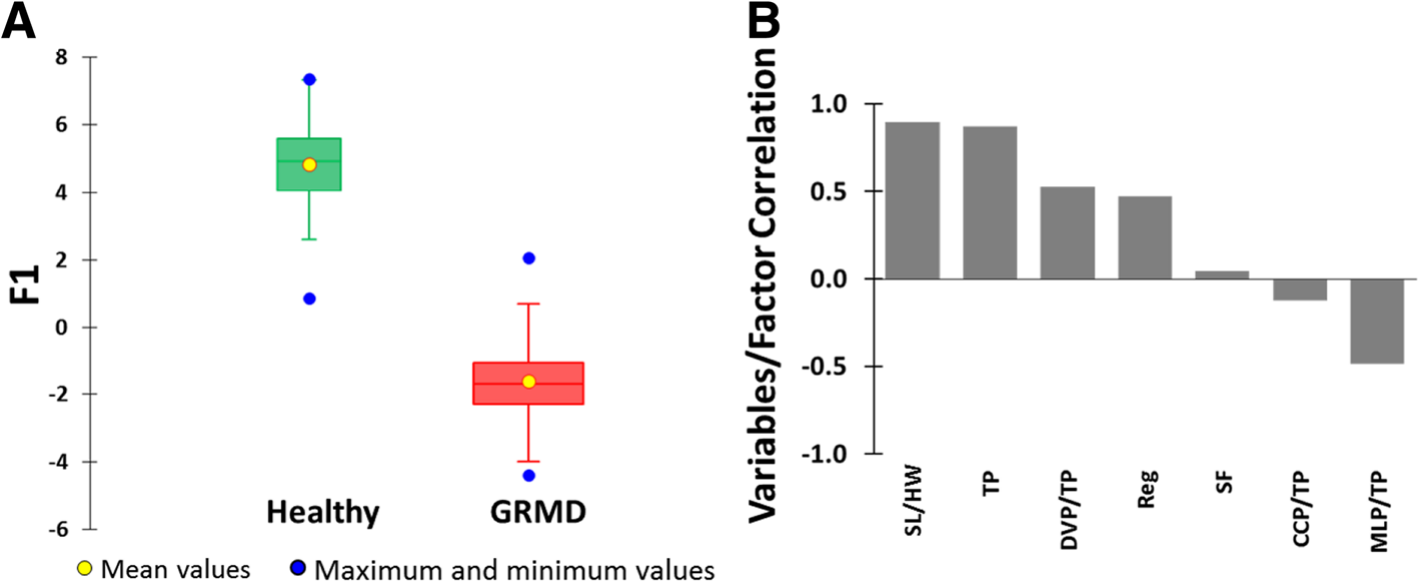
LDA Model 1: analysis of gait accelerometry parameters in healthy and GRMD dogs **a** Box and whisker diagrams of canonical variable F1 coordinates calculated by linear discriminant analysis of gait accelerometry parameters in healthy (*green*) and GRMD (*red*) dogs with genotype as the dependent variable. **b** Factor loading chart. SF, stride frequency; Reg, regularity; TP, total power; CCP/TP, cranio-caudal power normalized to TP; DVP/TP, dorso-ventral power normalized to TP; MLP/TP, medio-lateral power normalized to TP; SL/HW, stride length normalized to height at withers

We first investigated whether LDA can distinguish the gait phenotype of GRMD dogs from that of healthy controls independently of age, as previously shown using PCA [11]. To this end, using the same variables previously analysed by PCA, we performed LDA with genotype (GRMD or Healthy) as the dependent variable. The characteristics of this first model (Model 1) are listed in Fig. 1. Because only two groups were included, only one canonical variable (F1) was calculated. As shown in the factor-loading chart (Fig. 1b), the main discriminant variables were TP and SL/HW (positively correlated with the canonical variable) and MLP/TP (negatively correlated with the canonical variable). GRMD and healthy dogs were highly discriminated using Model 1. The accuracy of the model was assessed using a leave-one-out cross-validation approach, which revealed a low rate of misclassification <1%.

**Fig. 2:**
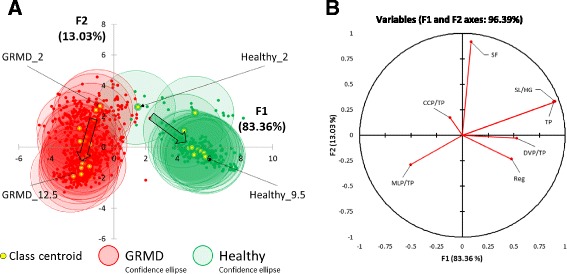
LDA Model 2: analysis of gait accelerometry parameters in healthy and GRMD dogs. **a** Linear discriminant analysis of gait accelerometry parameters for healthy and GRMD dogs with genotype and age (in months) as dependent variables. Individual measurements (*dots*) and groups (centroids and 95% confidence ellipses) are positioned on the plane using their values for the two first canonical variables, F1 and F2. Green and red colours correspond to healthy and GRMD dogs, respectively. For clarity, only the groups of younger and older animals were indicated for each genotypes. Arrows illustrate the evolution of class centroids according to age. The percentage variance explained by each canonical variable is indicated in parentheses. **b** Factor loading chart of F1 and F2 canonical variables. SF, stride frequency; Reg, regularity; TP, total power; CCP/TP, cranio-caudal power normalized to TP; DVP/TP, dorso-ventral power normalized to TP; MLP/TP, medio-lateral power normalized to TP; SL/HW, stride length normalized to height at withers

To better characterize gait alterations and improve the evaluation of treatment benefits at different disease stages, we expanded the dependent variable (GRMD or Healthy) by defining various age categories. Specifically, each group (GRMD and Healthy) was subdivided into age categories (expressed in months), for a total of 32 groups. As data were acquired twice monthly, the time interval classification was set to 0.5 months. Thus, data obtained for GRMD and healthy dogs were classified in groups GRMD_2 to GRMD_12 and groups Healthy_2 to Healthy_9.5, respectively. LDA was applied to this new scenario (Model 2). The results revealed that 96.4% of the variance was explained by the two first canonical variables (F1 and F2). The outcomes of Model 2 are shown in Fig. 2. Model 2 was less discriminant for gait phenotype than Model 1. Indeed, the 95% confidence ellipses calculated for GRMD dogs of up to 6.5 months of age partially overlapped with those calculated for the 2 month-old Healthy group (Fig. 2a). Accordingly, the rate of misclassification was very high (83.2%). The rate of misclassification was relatively low (1.2%) when phenotype was considered independently of age. The factor-loading chart (Fig. 2b) shows that F1 was mainly positively correlated with TP and SL/HW whereas F2 was mainly positively correlated with SF and negatively correlated with MLP/TP. All GRMD centroid coordinates were negative on the F1 axis, while all Healthy centroids were positive. F1 showed a high canonical correlation of 0.95, indicating that in Model 2, as observed in Model 1, F1 was discriminant for phenotype. For both the GRMD and Healthy groups, the centroid coordinates on the F2 axis were inversely proportional to age, indicating that F2 is associated with age and disease progression. Finally, a low canonical correlation of 0.78 was calculated for F2 in Model 2.

**Fig. 3:**
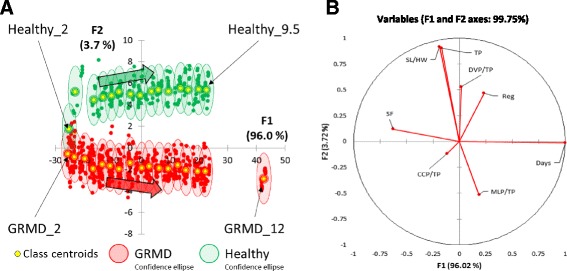
LDA Model 3: analysis of gait accelerometry parameters in healthy and GRMD dogs. **a** Linear discriminant analysis plot of gait accelerometry parameters for healthy and GRMD dogs with genotypes and age in months as dependent variables, and age in days as an additional explanatory variable. Individual measurements (*dots*) and groups (centroids and 95% confidence ellipses) are positioned on the plane using their values for the two first canonical variables, F1 and F2. *Green* and *red* colours correspond to healthy and GRMD dogs, respectively. For clarity, only the groups of younger and older animals were indicated for each genotypes. *Arrows* illustrate the evolution of class centroids according to age. The percentage variance explained by each canonical variable is indicated in parentheses. **b** Factor loading chart of F1 and F2 canonical variables. SF, stride frequency; Reg, regularity; TP, total power; CCP/TP, cranio-caudal power normalized to TP; DVP/TP, dorso-ventral power normalized to TP; MLP/TP, medio-lateral power normalized to TP; SL/HW, stride length normalized to height at withers

To improve the descriptive properties of the model while keeping its predictive capabilities, in addition to including age (in months) as a dependent variable, age (in days) was also included as an explanatory (or independent) variable in the LDA analysis. In this new model (Model 3), the two first canonical discriminant factors accounted for 99.7% of variance (Fig. 3). As expected, Model 3 was discriminant for data points reflecting age in days. In contrast to Healthy centroids, all GRMD centroids were negative on the F2 axis, indicating that F2 was discriminant for gait phenotype. The factor-loading chart (Fig. 3b) shows that F2 was predominantly and positively correlated with TP and SL/HW and negatively correlated with MLP/TP, whereas F1 was predominantly and positively correlated with age (in days) and negatively correlated with SF. After 2 months of age, no confidence ellipse overlap was observed between genotypes. While overlapping was still observed between confidence ellipses within each genotype group, this was limited to immediately contiguous age groups. The overall misclassification error for Model 3 was less than 12% and as low as 0.9% when phenotype was considered independently of age.

### Blind test for model validation

**Fig. 4:**
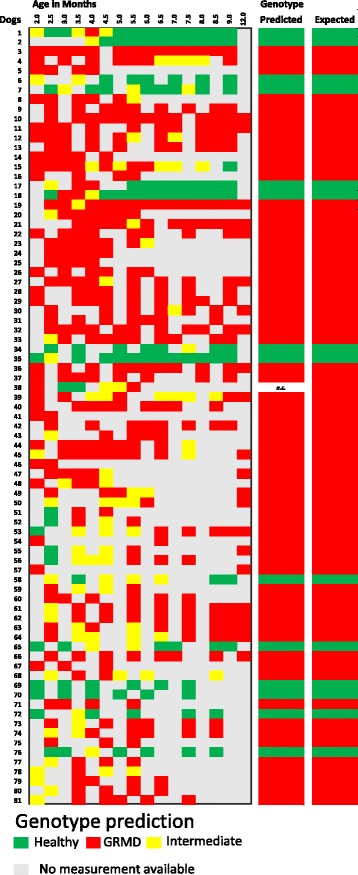
Gait phenotype separation: blind testing of LDA Model 3. To validate our proposed methodology, we used a blind dataset pertaining to 81 dogs and investigated the capability of Model 3 to predict genotype based on gait assessment. Each row corresponds to an individual dog and each column to a specific time point (age). Colours denote predicted phenotype based on the results of gait analysis. *Green*, *red*, and *yellow* correspond, respectively, to gait patterns that resemble that of healthy dogs (*p* > 95%), are strictly different to that of healthy dogs but similar to that of GRMD dogs (*p* > 95%), and are strictly different to the gait patterns of both healthy (*p* < 5%) and GRMD dogs (*p* < 5%). Grey colour indicates that measurements were not available. Cells of the penultimate column, entitled *Predicted*, are coloured to reflect the predominant phenotype (Healthy or GRMD) predicted by the model for each dog. Thus, *green* and *red* cells indicate that the gait of the corresponding dog resembles that of a healthy and a GRMD dog, respectively. *n.c.* denotes an inconclusive prediction result. Cells in the rightmost column are coloured according to the actual genotype of the dog

The models described in the present study were generated using accelerometric data from GRMD and healthy dogs from which measurements had been acquired every 15 days between 2 months of age and 9.5 (healthy dogs) or 12 (GRMD dogs) months of age. A dataset was generated as follows: numbers were randomly attributed, by an external scientist blinded to the data, to each of the dogs used to set up the model, and to a group of healthy and GRMD dogs for which only some measurements were available for the period of interest (2–12 months of age). Since all these animals were from the French GRMD colony, we used Model 3 to predict the gait type of each numbered dog at each time point for which data was available. The average probability of the dog belonging to the Healthy group was calculated for each time point and then summed. The following classification rule was applied: if the sum obtained was >0.95, the gait was considered that of a healthy dog, while if the value was <0.05, the gait was considered that of a GRMD dog. Values of between 0.05 and 0.95 were considered indicative of an intermediate gait phenotype. Finally, gait patterns were attributed to healthy or GRMD dogs, when the results for more than half of the time points corresponded to healthy or GRMD gaits, respectively. The results obtained are presented in Fig. 4. Of the 81 dogs tested, the predicted gait phenotype matched the actual phenotype and genotype in all cases but one, for which the results were inconclusive.

### Model validation

**Fig. 5:**
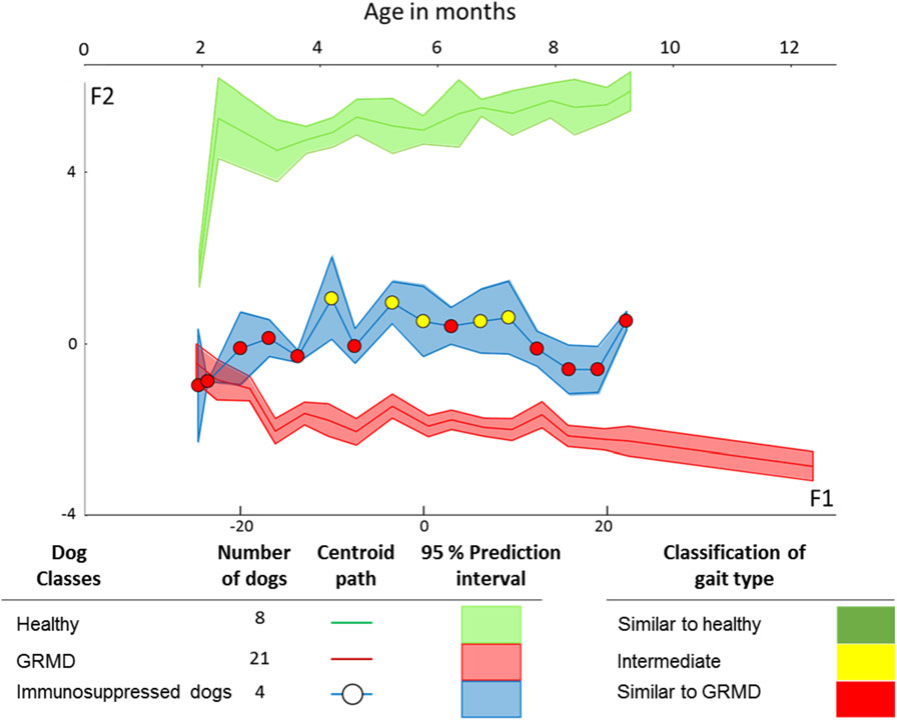
LDA Model 3 as a tool for evaluating the effects of immunosuppressive treatment on gait in GRMD dogs. Immunosuppressive treatment has beneficial effects on gait in GRMD dogs. Using Model 3, curves were generated by plotting centroids, and the associated 95% confidence intervals, corresponding to healthy, untreated GRMD, and immunosuppressant-treated GRMD dogs on F1 and F2 axes. Centroids corresponding to GRMD-immunosuppressed dogs are colour-coded to reflect their comparison with the healthy group: *green*, similar to healthy gait (*p* > *0.95*); *yellow*, intermediate gait (*0.95 > p* > *0.05*), *red*, similar to GRMD gait *(p < 0.05)*. For a more comprehensive representation, the projection of the age in days axe on the factorial plan was calculated and added as the upper axe (see text)

A previous study by some members of our group tested the effects of immunosuppressive treatments (oral administration of cyclosporine A and corticosteroids) on muscular dystrophy and overall health in GRMD dogs [12]. We reanalysed the experimental data from this previous study using our new methodology. Furthermore, we designed a new means of representing the outcomes that allows easier interpretation of the results (Fig. 5). We calculated the projection of the age (in days) axis and added it to the figure. In this way, the representation remains rigorous from a statistical point of view, showing F1 and F2 axes, but allows the reader to also refer to age of the dogs. Moreover, using the membership probabilities calculated by LDA, we applied a colour code to indicate on the graph to which gait type the data points should correspond: green, red, and yellow were applied to healthy, GRMD, and intermediate groups, respectively, as predicted by the assignment rule based on the mean of the probabilities (described above). This representation allows rapid assessment of the evolution of the gait of given dogs or group of dogs in comparison with that of other healthy or GRMD dogs. As shown in Fig. 5, immunosuppression in GRMD dogs had a significant beneficial effect on gait, although this was both partial and temporary.

## Discussion

We previously demonstrated that PCA, although a non-supervised analysis strategy, can distinguish the gait phenotype of GRMD dogs from that of healthy controls with reasonable accuracy, and does so independently of age [11]. We show in the present study that similar results could be obtained using LDA. However, when the age of the dogs were not take into account, the model built using LDA (Model 1) was found not to adequately represent the progression of the disease, which evolves over the course of the postnatal growth phase. On the other hand, addition of age, in months, as a supplementary dependent variable in LDA, the model obtained (Model 2) exhibited a very high rate of misclassification. This likely reflected the slow rate of disease progression over the short age intervals analysed.

Nonetheless, in Model2, the discriminant axis mainly associated with age and disease progression, F2, presented a low canonical correlation, indicating that the model was not sufficiently sensitive to accurately evaluate the impact of progressive dystrophy on gait in growing GRMD dogs. One means of improving the model was to increase the age interval (e.g. from 0.5 to 1 month), which would certainly reduce the misclassification error, but would also decrease the sensitivity of the model. The aim of this study was not to demonstrate altered gait in GRMD versus healthy dogs, but rather to develop a method to better identify gait alterations and their progression with age in GRMD dogs. To improve the descriptive properties of the model while keeping its predictive capabilities, we have artificially stretched the model (Model 3) in the direction of the progression of age, by introducing age (in days) as an explanatory (or dependent) variable in the LDA analysis, in addition to including age (in months) as a dependent variable. The dogs included in the study were not born on the same day. Additionally, sometimes some dogs were not able to walk, thus their acquisition session was delayed. By contrast, acquisition sessions were always done on the same day of the week, every 15 days. Thus, the age of the animals in days in the same age group in months could vary from about 7 days. The intrinsic variability of the age in days spread the model in the factorial plan helping to discriminate the different groups of age, although it could be assumed that this variability does not significantly impact the measurements. In Model 3, the two first canonical discriminant factors, F1 and F2, accounted for 99.7% of variance. F1 was predominantly correlated with age whereas F2 was discriminant for gait phenotype. Model 3 presented a low overall misclassification error and high canonical correlations of 0.99 and of 0.95 were calculated for F1 and F2, respectively.

In order to investigate further the suitability of Model 3 as a tool for gait analysis in preclinical studies with GRMD dogs, we used it to predict the genotypes of a cohort of 81 healthy and GRMD dogs starting from their gait accelerometry characteristics. For each dog of the cohort, a number was randomly attributed by an external scientist blinded to the data. Using this approach, when “the data were unblinded”, the predicted gait phenotype matched the actual phenotype and genotype in all cases but one. These findings strongly support the robustness and accuracy of the method.

Some members of our group tested, in a previous study, the effects of immunosuppressive treatments (oral administration of cyclosporine A and corticosteroids) on muscular dystrophy and overall health in GRMD dogs [12]. Indeed, immunomodulatory treatments have been employed in several studies assessing the effectiveness of gene, cell, or pharmacological therapies in dog models of DMD to supress the immune response to the viral vector, donor cells, and/or the transgene product [18–20]. The obtained results vary considerably depending on the variables measured. Although Barthélémy and colleagues [12] reported a more severe disease progression in terms of isometric force and histology, they also found an improvement in gait classification using a PCA-based gait index calculated from the seven accelerometric variables used in the present study. This longitudinal analysis was complex, as PCA, unlike DA, is an unsupervised approach. The authors performed PCA for each age category and calculated the corresponding Euclidean distance for each GRMD dog to the centroid of age-matched healthy dogs, and, thereafter, plotted the evolution of this distance with age. Using Model 3, we found that immunosuppression in GRMD dogs had a significant beneficial effect on gait, although this was both partial and temporary. This finding was in line with those of Barthélémy and coworkers [12]. Furthermore, LDA revealed that significant effects of treatment on gait observed at 4 months of age were preceded by signs of improvement that were evident as early as 3 months, supporting the sensitivity of our model.

## Conclusion

Using LDA we have generated a highly sensitive model of gait alterations due to muscular dystrophy in the GRMD dog. Our model shows a high degree of discriminatory accuracy, distinguishing the gait phenotype of GRMD dogs from that of healthy dogs as early as 2.5 months of age, and thus overcoming some of the difficulties in analysing a progressive disease that occurs during the growth phase of postnatal development. Moreover, we designed a new means of representing the outcomes of our analysis that allows for easier interpretation of the results. This is a key strength of our study: because preclinical results are the base upon which phase 1/2 clinical trials in human patients are prepared and designed, preclinical outcomes should be completely understandable to all those involved in the design and the testing of potential treatments from preclinical through to clinical phases. In our experience, graphical displays depicting only the two variables (F1 and F2) are very likely to be unclear, and sometimes misleading, to practitioners unfamiliar with DA or PCA.

We previously demonstrated that accelerometry combined with PCA constitutes a reliable follow-up tool for gait analysis in preclinical therapeutic trials using GRMD dogs. However, this approach has some limitations, including limited sensitivity, and high complexity of the data generated. The model presented here greatly improves upon this method by employing LDA.

## Abbreviations

CCP: Cranio-caudal power
DA: Discriminant analysis
DMD: Duchenne muscular dystrophy
DVP: Dorso-ventral power
GRMD: Golden retriever muscular dystrophy
HW: Height at the Withers
LDA: Linear discriminant analysis
MLP: Medio-lateral power
PCA: Principal component analysis
QDA: Quadratic discriminant analysis
Reg: Regularity
SF: Stride frequency
SL: Stride length
TP: Total power
